# Supplementary figures and images for: Linking Copper-Associated Signal Transduction Systems with Their Environment in Marine Bacteria
Source: Microorganisms. 2023 Apr 13;11(4):1012. doi: 10.3390/microorganisms11041012 (PMC10141476; doi:10.3390/microorganisms11041012)

Title: E value evaluation of representative organisms

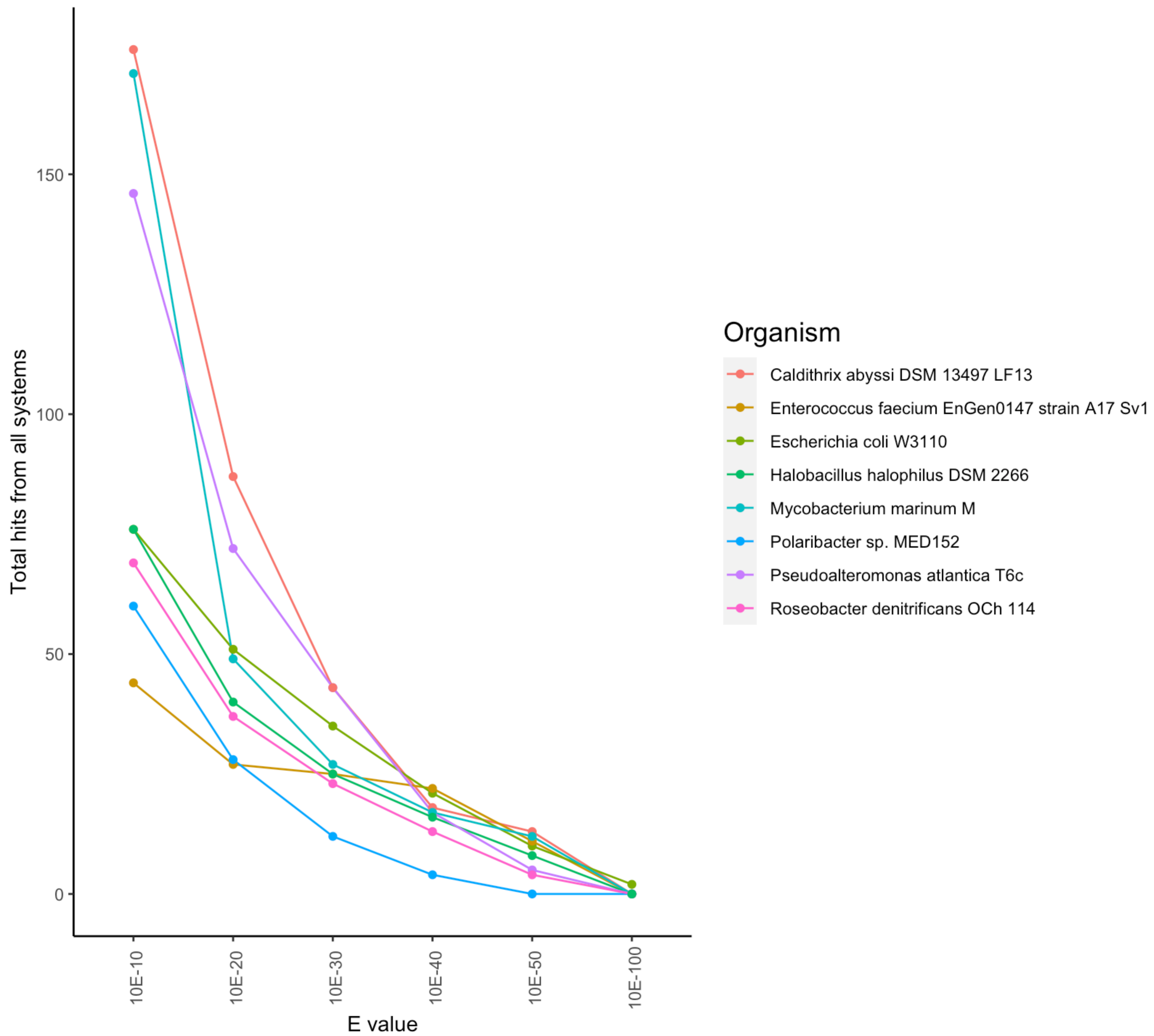

Supplement: Supplementary file 1 [file microorganisms-11-01012-s001.zip › Suplemental/Figure_S1.pdf]
